# A Survey of the Concept of Disturbance in Quantum Mechanics

**DOI:** 10.3390/e21020142

**Published:** 2019-02-02

**Authors:** Ernesto Benítez Rodríguez, Luis Manuel Arévalo Aguilar

**Affiliations:** Facultad de Ciencias Físico Matemáticas, Benemérita Universidad Autónoma de Puebla, 18 Sur y Avenida San Claudio, Col. San Manuel, Puebla 72520, Mexico

**Keywords:** disturbance, quantum mechanics, uncertainty principle, information gain

## Abstract

The concept of disturbance is of transcendental importance in Quantum Mechanics (QM). This key concept has been described in two different ways, the first one considering that the disturbance affects observables like *x* and *p*, as in the Heisenberg’s analysis of the measurement process and the other one takes into consideration that disturbance affects the state of the system instead. Entropic information measures have provided a path for studying disturbance in these both approaches; in fact, we found that initially it was studied by employing these entropic measures. In addition, in the last decade, there was an extensive amount of analyses and several new definitions of the disturbance concept emerged. Many crucial factors like this have inspired this concise paper which gathers the different concepts and definitions that have emerged through time for the better understanding of this topic.

## 1. Introduction

The key concept of disturbance is inherent in Quantum Mechanics (QM) due to the nature of the systems and the nature of the observer. In this regard, one of the most significant examples of the importance and the necessity for a good definition of disturbance is given by the security of the BB84 scheme on quantum cryptography. In this case, the public test certifies that none of the four states that a sender sent was disturbed, "but any measurement which fails to disturb nonorthogonal states also yields no information about them” [1]. The key point in the demonstration of the statement of Bennett et al. is in the fact that, if there is no a disturbance, then the eavesdropper system is left in the same state after interacting with the state sent, i.e., the eavesdropper does not acquire any information [1].

On the other hand, the concept of disturbance represents one of the striking characteristics of how QM differentiates from classical mechanics. Indeed, the development of QM in the early years of its conception was driven by the idea of getting to know the microscopic world and, consequently, understand the effects that the classical theory could not explain. In this regard, it was believed that the interaction between the observer and the observed could not exist without a disturbance due to the difference of size (It is a fact that the difference of proportions between us and quantum systems is definitive to the interaction, but here we do not discuss about scales of validity for the quantum theory. This is an issue which is absolutely left aside in classical mechanics), even though recent works describe mesoscopic systems (or even macroscopic) employing quantum theory with very interesting results. For a deep discussion, see [2].

The existing disturbance in the interaction was stated by Born [3,4], while discussing the statistical interpretation of quantum mechanics in 1929. Here, the probabilities to find certain outcomes in a measurement process were related to the fact that there are different possible results when one wants to know a property of a system. That is, there is not certainty about the result that one would find; instead, in the quantum world, we lose our power of predictability.

Heisenberg was the first in getting the insight that a measurement process affects the quantum systems by causing a kind of disturbance; however, he never provided a precise definition of it. Astonishingly, it was only in the last few decades when an effort to formally define the concept of disturbance was initiated. To the best of our knowledge, one of the earliest efforts that formally define the disturbance caused by the measurement process was given by Srinivas [5], who gives an estimate of the disturbance, when measuring one observable by using the joint probability of a sequence of measurement as it was proposed by Wigner [6], where he deduced a lower bound for this definition of disturbance.

On the other hand, it was Fuchs who pointed out that one of the fundamental contributions of quantum cryptography was to focus on the state of the quantum system in order to address an information–disturbance trade-off [7], releasing disturbance from being gauged in terms of observables. Additionally, it is worth mentioning that Matteo G. A. Paris [8] warned us about the use, without caution, of fidelity in quantum information because inclusive states that have a high amount of fidelity differ substantially in their properties. Furthermore, Kochen and Specker [9] pointed out that observables do not possess a predetermined previous value; this puts restrictions on the available tools for defining disturbance in the case of observables’ projective measurement. All the previous considerations are highly important when gauging disturbance, and a brief mention of them is given in the next section.

There is no universal way of defining disturbance; its definition highly depends on the situation studied. However, generally speaking, the concept of disturbance is associated with the process of measurement in at least three ways: (i) noise-disturbance, which captures the notion that there is trade-off between the noise in getting the value of an observable and the disturbance induced, (ii) information–disturbance, which captures the trade-off between the extraction of information and the disturbance caused, and (iii) disturbance–disturbance, which captures the trade-off of disturbing the statistical distribution of a given initial state.

Hence, the main aim of this survey is to collect the definitions of disturbance in quantum mechanics that have been stated in literature in recent years. One of the goals of the studies reported in literature is to maximize the information extraction while reducing the disturbance caused; another goal is to reduce the noise and to reduce the disturbance. We do not intend to be exhaustive, so this is a brief review intended to show the different facets of disturbance in quantum mechanics. We apologize for the omission of many excellent works not directly mentioned. Furthermore, our intention is to get the attention of researchers, textbooks editors and teachers towards these new developments in quantum mechanics.

This paper is structured as follows: in Section 2, we give some definitions. In Section 3, we describe the concept of disturbance in the information–disturbance trade-off, whereas, in Section 4, we expose the disturbance in the framework of the noise-disturbance trade-off, where an evident differentiation of understanding of the concept exists. Finally, in Section 5, we present a brief discussion and our conclusions.

## 2. Definitions

In this section, we introduce some quantities and definitions that will be of use in the following sections.

### 2.1. Uncertainty Principle

The uncertainty principle in QM was proposed by Werner Heisenberg in 1927 [10], since that time, the principle has been enhanced, modified and extended, arriving to three main accepted uncertainty principles in QM. For a more in-depth discussion of this topic, see Reference [11]. The best known principle is the Heisenberg Preparation Uncertainty Principle, which shows the impossibility to prepare a quantum system with two properties that corresponds to two non-commuting observables, which can be well known at the same time. The Heinseberg Uncertainty Principle is a consequence of the formalism of the quantum theory only and it is not directly related to the act of measurement, namely, this facet of uncertainty in QM is inherent in the theory. On the other hand, the disturbance is ubiquitous in the measurement process; furthermore, uncertainty is the result of the disturbance inflicted by the measurement [12,13].

Each of the three uncertainty principles mentioned above have their own uncertainty relation [11]. A direct connection between the statement of the principle and the mathematical relation is stated in Reference [11]. These three statements are the following:

1. Preparation Uncertainty Principle: It is impossible to prepare states whose two incompatible properties, for example position *x* and momentum *p*, are well determined at the same time.
2. Uncertainty Principle of Simultaneous measurements: It is impossible to measure two incompatible properties of a state at the same time.
3. Noise–Disturbance Uncertainty Principle: It is impossible to measure a property of a state without disturbing another property of the state.

As we can see, the only principle that discusses directly the disturbance is the third principle; and it is considered as a first approach to describe disturbance in QM. In this way, it is possible to distinguish the disturbance by using different criteria and consequently study it from many points of view. Hence, in this paper, we differentiate the disturbance by identifying the different relations that exist between disturbance–information and disturbance–noise; we also recognize the characteristics of the system’s description that were subjected to the disturbance. We choose this way to classify the disturbance due to the diversity of the existing works about this topic and as a starting point to suitably sort the different definitions that have appeared in the recent years.

It is important to remember that the uncertainty principles and uncertainty relations have been the topic of many research articles. In particular, the area of Entropic Uncertainty Relations (EUR) is a leading branch in the study of uncertainty relations [13,14,15,16,17,18,19,20,21]. The study of EUR has given rise to current definitions of uncertainty and disturbance in quantum mechanics. Such definitions are given in terms of entropy, a quantity related to thermodynamics, but carried to different scientific areas in the 20th century. The area of Information Theory (IT) is one of the areas where the concept of entropy has had a deep impact and a close connection between QM and IT has emerged in the last few decades. Such entailment has had significant implications in the concept of disturbance and its relation with information has a paramount importance in these days.

### 2.2. The Bell–Kochen–Specker Theorem

When we talk about disturbance in the state or observables of a system, we remember almost instantly the Bell–Kochen–Specker Theorem (BKST) [9,22,23], which shortly says that the observables of a quantum system do not possess a predetermined value before the act of measurement. This theorem is also important to our study due to the constraints that it imposes to disturbance.

### 2.3. Fidelity

Because the fidelity is broadly used to define disturbance, in this subsection, we briefly review it. The Fidelity F of quantum states was first defined in terms of the transition probability by Uhlmann [24] and subsequently analyzed by Jozsa [25]. These authors propose the fidelity as the generalization of the transition probability for pure states to the case of mixed states.

For pure states, the fidelity is reduced to the transition probability and it has a simple physical interpretation as the probability that $\rho_{2}$ passes the test of being $\rho_{1}$; for mixed states, it can be thought as a measure of distinguishability [25], this interpretation comes from the representation of F as a purification procedure, i.e., $F(\rho_{1},\rho_{2})=\max|\langle \psi_{1}|\psi_{2}\rangle |$, where $|\psi_{1}\rangle$ is the purification of $\rho_{1}$ and $|\psi_{2}\rangle$ is the purification of $\rho_{2}$.

The fidelity F is mathematically defined as:

(1) $$F\left(\rho_{1},\rho_{2}\right)={\left\{tr{\left(\sqrt{\rho_{1}}\rho_{2}\sqrt{\rho_{1}}\right)}^{1/2}\right\}}^{2}.$$

It is worth mentioning that there are many uses for the name fidelity; for example, some authors use $\sqrt{F}$ as the fidelity. On the other hand, the fidelity has interesting and desirable good characteristics such as, among others: (i) being bounded, that is $0\leq F(\rho_{1},\rho_{2})\leq 1$, $F(\rho_{1},\rho_{2})=1$ iff $\rho_{1}=\rho_{2}$, while $F(\rho_{1},\rho_{2})=0$ iff $\rho_{1}$ and $\rho_{2}$ are orthogonal, (ii) is symmetric, i.e., $F\left(\rho_{1},\rho_{2}\right)=F\left(\rho_{2},\rho_{1}\right)$; and (iii) is concave, i.e., $F\left(\rho ,a\rho_{1}+\left(1-a\right)\rho_{2}\right)\geq aF\left(\rho ,\rho_{1}\right)+\left(1-a\right)F\left(\rho ,\rho_{2}\right)$ for a∈[0,1].

Several bounds have been found for the fidelity, for example $F\left(\rho_{1},\rho_{2}\right)\leq \left(tr\rho_{1}\right)tr\left(\rho_{2}\right)$. One important result is the following upper and lower bounds demonstrated by Miszczak et al. [26]:

(2) $$F\left(\rho_{1},\rho_{2}\right)\leq tr\rho_{1}\rho_{2}+\sqrt{\left(1-tr\rho_{1}^{2}\right)\left(1-tr\rho_{2}^{2}\right)},$$

and

(3) $$F\left(\rho_{1},\rho_{2}\right)\geq tr\rho_{1}\rho_{2}+\sqrt{{\left(tr\rho_{1}tr\rho_{2}\right)}^{2}-tr\rho_{1}\rho_{2}\rho_{1}\rho_{2})}.$$

The upper bound is called the super-fidelity and the lower bound is called the sub-fidelity [26].

It is worth mentioning that, despite being so widely used, recently, some concerns have arisen about the use of fidelity for assessing quantum correlations; in particular, it has been showed that two states can possess high fidelity, of the order of F≈9.25; however, while one of them is an entangled state, the other one is a separable state, hence possessing quite different physical properties [8]. This was also proven for continuous variable systems [8,27]; see also [28,29].

### 2.4. Measurement and Entropy

Many proposals for defining disturbance are made under the Generalized Measurement approach; in this case, there exists a set of measurement operators $M_{m}$ which represent the probabilities p(m) and the output state $\rho_{f}$ as follows:

(4) $$p\left(m\right)=tr\left\{\rho_{i}M_{m}^{†}M_{m}\right\},$$

and

(5) $$\rho_{f}=\frac{M_{m}^{†}\rho_{i}M_{m}}{p(m)},$$

where $\rho_{i}$ is the initial state.

On the other hand, the conditional entropy is defined as:

(6) $$S\left(B|A\right)=-\sum_{a\in A,b\in B}p\left(a|b\right)\log\left(\frac{p(a,b)}{p(a)}\right),$$

where *A* and *B* are any two events, *a* and *b* are the possible outcomes of the events, p(·|·) is the contidional probabliity and p(·,·), the joint probability.

The joint entropy is defined as

(7) $$S\left(A,B\right)=-\sum_{a\in A,b\in B}p\left(a,b\right)\log\left(p(a,b)\right),$$

for *A* and *B* two events and p(·,·) the joint probability.

Another important definition is that of the von Neumann Entropy

(8) S(ρ)=−(ρlogρ),

with ρ a density operator and the usual trace.

Finally, the Jensen–Shannon Entropy is defined as [30]:

(9) $$D_{PQ}=\sqrt{\sum_{i=1}^{N}(p_{i}\ln\frac{2p_{i}}{p_{i}+q_{i}}+q_{i}\ln\frac{2q_{i}}{q_{i}+p_{i}}}),$$

with $p_{i}$ and $q_{i}$ two probability distributions.

## 3. Disturbance in the Information–Disturbance Trade-off (IDT)

It is indisputable the prominent position that the Information Theory (IT) has acquired in the development of science and technology, for instance, the linkage with QM has been clearly seen for decades. The adoption of the Shannon Entropy as a measure of information in the decade of the forties in the previous century holds a notorious place in the history of science [31] and represents a starting point for the development of communication sciences [12]. Certainly, the concept of disturbance has been studied under the framework of IT, given us new definitions and trade-off relations.

Particularly, the disturbance has been a tool for the practical application of the uncertainty principle in the quantum cryptography area, where it is believed that encoding information in qubits could help to protect the information from eavesdropping. In fact, this idea is based on the point that the theory of measurement in QM predicts an unavoidable disturbance of the quantum state when the system interacts with the measurement apparatus.

Before reviewing the Information–Disturbance Trade-off (IDT), let us recall the previous and closely related Information-Information trade-off due to M. J. W. Hall [32], who analyses the case of gaining information of an observable *A* only at the expense of the information of complementary observables. Hall calls this phenomenon the Information Exclusion Principle (IEP), this principle predicts inequalities of the following form

(10) $$\sum_{l}I\left(A_{l}|\varepsilon \right)\leq J\left(A_{1},A_{2},\ldots A_{l},\ldots ,\rho_{\varepsilon }\right),$$

where $I\left(A_{l}|\varepsilon \right)=S\left(A|\rho_{\varepsilon }\right)-\sum_{i}p_{i}S\left(A|\rho_{i}\right)$ is the mutual information given in terms of the conditional entropy (6) and $J(A_{1},A_{2},\ldots A_{l},\ldots ,\rho_{\varepsilon })$ is a non-trivial bound, we have $\rho_{\varepsilon }$ as the density operator for an ensemble ε and $p_{i}$ are the probabilities of each $\rho_{i}$. Equation (10) is considered by Hall as an information theoretic analog of Heisenberg uncertainty relation, and is one of the oldest entailments between IT and QM, as well as one of the first reformulations of the uncertainty principle.

From Hall’s definitions, we can interpret the loss of information as the disturbance. For example, in the case of spin 1/2 particles $I\left(\sigma_{1}|\varepsilon \right)+I\left(\sigma_{2}|\varepsilon \right)+I\left(\sigma_{3}|\varepsilon \right)\leq \log2$. Therefore, we can attain the maximum information for $I(\sigma_{1}|\epsilon )$, namely $I(\sigma_{1}|\epsilon )=\log2$, only at the expense of $I(\sigma_{2}|\varepsilon )$ and $I(\sigma_{3}|\varepsilon )$.

On the other hand, taking Reference [33] as our basis, it is possible to discern the following two approaches into IDT: an Information Theoretic approach, which here we will refer to it as Informational IDT, and an Estimation Theoretic approach, which here we will refer to it as Estimation IDT. The former approach is based on entropic quantities, whereas the latter is usually stated in terms of fidelity [25], and its goal is the estimation of parameters. The main attribute of the IDT is the study of the system by focusing on the evolutions or alterations on the state of the system, a quality that is given thanks to their inherent relation with IT [12].

### 3.1. Informational IDT Approaches

Starting with the Informational IDT approach, a crucial paper by Fuchs and Peres [34] shows for the first time the trade-off relation between information and disturbance; in this paper, they propose one of the earliest definitions of disturbance [34]. The problem they were concerned about is with the amount of information that an observer can extract from a quantum system and how this action disturbs the system; this problem is related to the act of sending and receiving a message considering the possibility of a third party intromission, which wants to get information from this message. The Informational IDT that they introduce is defined by considering the information gain as the mutual information [32,34].

The definition of disturbance proposed by Fuchs and Peres is given in terms of the discrepancy rate (which in this case corresponds to the fidelity F defined in Section 2), which is defined in terms of the mean value as:

(11) $$D=1-\langle \psi_{n}|\rho_{n}|\psi_{n}\rangle .$$

The interpretation of disturbance related to Equation (11) is associated with the distinctness that the receiver is able to detect a change in the state sent owing to the influence of a third party. This can be seen in Equation (11), where $\rho_{n}$ is the state after the eavesdropper interaction and $|\psi_{n}\rangle$ is the state sent by the sender. It is valuable to mention that this paper can be considered as a starting point to the Informational IDT procedures [7,25,35]. This point of view is shared by many authors, not necessarily in the Informational IDT [36]. To the best of our knowledge, the union of the concept of information and disturbance was accomplished for the first time here, leading to the current situation where the trade-off between them is a common topic of study. In this interpretation, the lower bound $D_{0}$ that disturbance can attain when an amount of information is extracted is given by:

(12) $$D_{0}=\frac{\epsilon^{4}\left(S^{2}-S^{4}\right)}{16},$$

where S=〈0|1〉=sin2α is the scalar product of the non-ortogonal states used by the sender and ϵ is an angle parameter; see Reference [34] for details.

Another important course of action towards the definition of disturbance in the Informational IDT approach was given by D’Ariano in his article of 2003 [20]. D’Ariano based his definition on the probability of undoing a generalized measurement; hence, he defines disturbance as:

(13) D(M)=1−C(M).

Here, the disturbance of the state in Equation (13) is given by the fidelity, where C(M) is the input-output unitary correlation of *M* defined as the fidelity between the initial state |ψ〉 and the final state V|ψ〉. According to D’Ariano, an attribute of the disturbance is that the disturbance itself would be a function of the probabilities of reversing the effect it produces on the system, which is a requirement that had been studied deeply by other authors [12,37]. For the singular value decomposition $M=X_{M}\sum_{M}Y_{M}^{†}$, the disturbance is minimum when the square of the singular value of *M* fulfills $\sigma_{i}^{2}\propto \lambda_{i}^{-1}$. As stated in the introduction, this extreme is of great interest because the minimum of the disturbance corresponds to a maximum information gain.

By following the approach of D’Ariano [20], Maccone studies the implications of defining disturbance as an irreversible change in the state of the system. Here, it is proposed a series of requirements that the disturbance should fulfill in accordance with Reference [20]. Maccone presents a definition of disturbance that has two mechanisms of state change, the dynamics of the system and the acquisition of information from the system. In this article, this definition of disturbance is expressed by the following quantity:

(14) $$D=S\left(\rho \right)-I_{c}.$$

In Equation (14), S(ρ) is the von Neumann entropy, given by Equation (8), and $I_{c}=S\left(Q\left(\rho \right)\right)-S\left(\left(Q⊗I|\psi \rangle \langle \psi |\right)\right)$ is known as the coherent information [38], taking |ψ〉 as a purification of ρ and *Q* corresponding to the map that describes the evolution of the measurement apparatus in the sense established by Kraus [39]. Equation (14) is a definition that was explicitly made to count the invertibility of the measurement. Thus, this article is taken as a first attempt to quantize the relations between the information and disturbance [40]. In this approach, the information–disturbance trade-off is given by I≤D; hence, the minimum disturbance is reached when the information equals the disturbance, this occurs when the measurement maps the different vectors of the initial state into orthogonal subspaces.

Following this line, the article of F. Buscemi, M. Hayashi and M. Horodecki of 2008 [40] is a reformulation of the seminal idea of Maccone [37]. Here, they have as a main goal to research the IDT by using quantum entities; in particular, the definition of disturbance is related to undoing the state change due to a measurement. The measure of disturbance is given by the following equation

(15) $$D=S\left(\rho \right)-I_{c}^{\prime },$$

where $I_{c}^{\prime }$ is the coherent information, a function of the Von Neumann entropy. This definition of disturbance generalizes the notion of coherent information loss for quantum channels. Notice the difference between Equation (14) and Equation (15). In addition, take into account that, in this paper, $I_{c}^{\prime }$ is a function of genuine quantum entities and the definition of disturbance of Equation (15) is customized for general measurements. Under this perspective, the minimum disturbance is reached when it equals the quantum information gained.

### 3.2. Estimation IDT Approaches

The Estimation IDT approaches are well known for their application to the study of the IDT by using the fidelity. In this sense, they serve as a means to estimate the parameters that describe information and disturbance.

In this approach, we have the article of C. A. Fuchs of 1998 [7], where it was exposed clearly and for the first time the idea of Estimation IDT. Fuchs proposed the use of the fidelity as a measure of information and disturbance; in particular, defining the disturbance as the average fidelity between the input $\rho_{s}$ and the output $\rho_{s}^{A}$ states

(16) $$D=1-\frac{1}{2}\sum_{s}F\left(\rho_{s},\rho_{s}^{A}\right).$$

In this equation, $F(\rho_{s},\rho_{s}^{A})$ is the fidelity defined in Section 2 [25]. As a preliminary for Estimation IDT relations, the article of Fuchs is open to a wide diversity of measures of disturbance because the arbitrariness of choosing such measure is due to the broad range of applications for the system. In this case, the minimum disturbance $d_{0}$ attained is given by $d_{0}=\frac{1}{2}-\frac{1}{2}\sqrt{1-S^{2}+S^{4}}$, where again *S* is the scalar product of the non-orthogonal state.

Following the open path by Fuchs, there are two papers written by H. Barnum in 2001 and 2002 [35,41], where he studies the relation between information and disturbance. The given treatment that Barnum proposes is hybrid because it describes the disturbance in terms of fidelity and the information is described as the mutual information. In his articles, Barnum also explores the frontier of the IDT, namely the limit of minimal disturbance for the greatest amount of information. The main measure of disturbance that is calculated here is

(17) $$D=1-F(\rho ,\sum_{j}A_{j}\left(\rho \right))$$

for $F\left(\rho ,\sum_{j}A_{j}\left(\rho \right)\right)={\left(\sqrt{\rho^{\frac{1}{2}}\left(\sum_{j}A_{j}\left(\rho \right)\right)\rho^{\frac{1}{2}}}\right)}^{2}$; that is, the fidelity in terms of the Josza [25] definition, where $\sum_{j}A_{j}\left(\rho \right)$ are measuring operators and ρ is the initial state of the ensemble. Barnum also proposes another measure for the disturbance, in this case, related with the entanglement fidelity [42]

(18) $$D_{e}=1-F_{e}\left(\rho ,A\right).$$

Equation (18) gives the disturbance as a function of $F_{e}\left(\rho ,A\right)=\sum_{k}{\left|A_{k}\rho \right|}^{2}$. Here, ρ is an initial bipartite state and $A_{k}$ is a bipartite measurement operator. The entanglement is a common tool for quantum tasks, and its presence in the description of disturbance is remarkable.

On the other hand, Banaszek in a remarkable article of 2001 [43] describes the estimation quality of the state of a system (that in turn corresponds to the information gained) and the degree in which the initial state has been altered (disturbed) by the measurement. The IDT is studied for the case of a single copy of the system employing the fidelities *F* and *G*, currently known as operation fidelity and estimation fidelity, respectively [44,45]. The disturbance is measured by means of the fidelity,

(19) $$F=\int d\psi \sum_{i=1}^{N}{\left|\langle \psi |{\hat{A}}_{i}|\psi \rangle \right|}^{2},$$

where ${\hat{A}}_{i}$ stands as a measurement operator for the outcome *i* and |ψ〉 is the initial state of the system. This definition measures the disturbance by using the mean value of the measured observables to display the difference between the initial and the final states. In this case, the optimal information that is retrieved corresponding to the minimum of the disturbance occurs when the information equals the disturbance and both of them reach the amount 2/(d+1), where *d* is the dimensionality of the particle. This article is a milestone for the Estimation IDT approaches and the use of fidelity for describing the IDT.

The main idea of Banaszek was improved by himself and Devetak in the same year of 2001 [46]. In this paper, the authors explore a generalization of the Estimation IDT given by the fidelity trade-off for an ensemble. This paper explains the exchange between the estimation fidelity *G* and the operation fidelity *F*. As we see from Reference [43], the operation fidelity is related to the disturbance, but, in Reference [46], the definition is connected with a qubit ensemble, as made evident in the expression for F,

(20) $$F=\int d\Omega \sum_{i=1}p_{i}\left(\Omega \right)\langle \Omega |{\hat{\rho }}_{i}^{red}\left(\Omega \right)|\Omega \rangle |\Omega \rangle .$$

In Equation (20), ${\hat{\rho }}_{i}^{red}$ is the reduced density matrix over *N* qubits and $p_{i}\left(\Omega \right)$ is the probability for obtaining the outcome *i* for an ensemble of *N* qubits, each one prepared in a pure state |Ω〉.

Years later, in 2006, M. F. Sacchi [44] quantified the disturbance by using the operation fidelity, which measures the similarity of the state of the system before and after the measurement. The trade-off between information and disturbance is studied for a maximal entangled state, in this respect, the disturbance proposed follows this idea. The disturbance is defined in the following equation:

(21) $$D=\frac{1-F}{1-F_{min}}.$$

Notice that this is given in terms of the fidelity of Equation (19), but taking the definition for a bipartite state and a measurement operators. $F_{min}$ is the average fidelity with the maximally chaotic state, i.e., the minimum fidelity. Due to their possible application to quantum cryptography, this paper focuses on maximally entangled states. By using entangled states, Sacchi improves the disturbance given by Banazek [43], and he found that, in the same way as in previous works, the fidelity equals the estimation.

Interestingly, the information–disturbance trade-off was extended to include continuous variable systems; for example, in 2006, M. G. Genoni and M. G. A. Paris presented a scheme to quantify both the information gain and the disturbance by coupling the state of the system with two probe systems [45]. In this regard, the model they use is that of the generalized measurement; they also explore different ranges of energies on the signal and the probe. Here, they defined disturbance in terms of the transmission fidelity as:

(22) $$F_{a}=\int dbq\left(b\right){\left|\langle \phi_{b}|\psi_{a}\rangle \right|}^{2},$$

where $|\phi_{b}\rangle$ is the estimated state and $|\psi_{a}\rangle$ is the initial state. They also found that the continuous variable systems give better trade-off than the discrete ones. This information–disturbance trade-off for continuous variables was later improved in a following work [47]; see also [48].

Another interesting point of view is given by Shitara, Kuramochi and Ueda in their article of 2016 [33]. In this paper, the information is related to the classical Fisher information and the disturbance is characterized in terms of the average loss of quantum Fisher information, where the disturbance is expressed as

(23) $$\Delta J_{\theta }^{Q}=J_{\theta }^{Q}-\sum_{j}p_{\theta ,j}{J^{\prime }}_{\theta ,i}^{Q}.$$

Equation (23) is defined as the difference between the initial and final Fisher information $J_{\theta }^{Q}$ and ${J^{\prime }}_{\theta ,i}^{Q}$, for an ensemble *Q*, with θ a parameter and $p_{\theta ,i}$ the probability related to the final state. Shitara et al. show that the lowest bound for disturbance is given by the classical Fisher information.

A new approach to the Estimation IDT was introduced in the paper by Sun et al. [49]. In this paper, the authors propose to use the information retrieved from a first measurement of an observable *A* to wipe out the bias in a sequential measurement of another observable *B*. Here, disturbance is understood as the bias induced by the first measurement. The authors also warn us that their definition could be best understood as precision rather than disturbance. Here, the measurement precision that is proposed is given by the following equation:

(24) $$I^{B}=\sum_{i}{\left(\frac{\partial \ln p_{B=i}}{\partial \langle \Phi |B|\Phi \rangle }\right)}^{2}\ln p_{B=i},$$

where $|\Phi \rangle =\sin\alpha {|0\rangle }_{s}+\cos\alpha e^{i\phi }{|1\rangle }_{s}$ is the more general qubit state. In this scenario, Sun et al. use the fact that any joint measurement can be implemented by a sequential measurement [50].

A paper by Seveso and Paris [51] worthy of mention analyses the trade-off between information and disturbance in the Estimation IDT approach by using four measures of disturbance: the already mentioned average information [33], the fidelity based disturbance [7], the τ-disturbance and the π-disturbance. As a consequence, they found interesting outcomes and they also give plots of the trade-off relations.

Finally, to end this section, we want to mention briefly the work of Hashagen and Wolf [52]. This article underlines the universalization of the IDT. They propose general measures for information and disturbance and they also study their relation of reciprocity. The importance of this article lies in the fact that it is a serious attempt to include all the IDTs in one formalism. This proposal reaches the boundaries of the topic and it gives new ways to study the IDT.

## 4. Disturbance in the Noise–Disturbance Trade-off (NDT)

### 4.1. Disturbance in the Noise–Disturbance Trade-off (NDT)

As it was discussed in the Introduction section, the idea that the disturbance can affect the observables of a system faces the fact that observables do not have predetermined values [13,23]. However, many of the best known definitions and applications of disturbance in QM have been built on this idea [14,15]. It is not a surprise that one of the first definitions of disturbance in the history of quantum mechanics appertain to this type of disturbance.

The article by Srinivas [5], as far as we are aware, is one of the first papers where a definition of disturbance can be found. This paper does not provide a literal definition of disturbance, but an intuitive idea of the concept is offered; we can see this point in the following excerpt, page 682:

“(...)Actually an estimate of the interference caused by an earlier A-measurement on the uncertainty in the outcome of a later B-measurement can be had from the following inequality:”

[5]

Notice that, for better understanding, the inequality that appears in the paper of Srinivas is rewritten to fit our nomenclature as follows:

(25) $$S_{A,B}^{\rho }\left(B\right)\geq \log\left\{\frac{1}{\sup\|\sum_{i}P^{A}\left(a_{i}\right)P^{B}\left(b_{j}\right)P^{A}\left(a_{i}\right)\|}\right\},$$

where $S_{A,B}^{\rho }$ is the Shannon entropy, *A* and *B* are operators, ρ is the state of the system, $P^{A}\left(a_{i}\right)$ and $P^{B}\left(b_{j}\right)$ are projection operators, and sup denotes the supremum over all of the eigenprojectors of *B*, all established in the Heisenberg picture.

As we can note from the previous quotation, the stated article [5] gives us an estimate of the interference that is found in the uncertainty of the observable *B* due to a previous measurement of *A*, as shown in Equation (25). This main idea has some similarities to what has been discussed in recent works about disturbance, for instance [21], where the comparison between the values that a measure of disturbance takes in two different moments is applied. However, quantifying the uncertainty in consecutive measurements was what Srinivas was really interested in; that is, when the measurement of an observable *A* is followed by the measurement of another observable *B*.

Later, using a similar procedure than in his previous paper, Srinivas himself found a stronger bound [53]

(26) $$S_{A,B}^{\rho }\left(B\right)\geq \log\left(\frac{1}{\sup_{ij}{\left|\langle a_{i}|b_{j}\rangle \right|}^{2}}\right),$$

the previous bound is greater than the Massen–Uffink lower bound. Additionally, Srinivas also extend the latter result to successive measurements for more than two observables.

An improved form of the Srinivas uncertainty relation was given by Back et al. [54]. They have shown that a lower bound for successive measurement is attained by the conditional entropy of the measured observables, i.e., H(A|B)≥−2lnc, which implicitly puts a lower bound to the disturbance. This analysis was also extended to cover the case of Positive Operator Valued Measure (POVM) in Reference [55].

On the other hand, Arthurs and Goodman in 1988 studied the concept of noise through the description of a generalized Heisenberg uncertainty relation [56]. The uncertainty relation in this case imposes a lower bound on the inherent extra noise in measurements and it demonstrates that the noise is due to the measuring process. The noise in measurement appears when the measuring apparatus is considered. To define this noise, they use a noise operator as follows:

(27) $$N=Y-G_{Y}A,$$

which describes the correlations between a tracker observable *Y* and the observable of the system *A*, where $G_{Y}$ is a real constant. The description that the authors gives is established on the Heisenberg picture; thereby, this description faces the constraints imposed by the BKST. This article is a reference for other authors that study the NDT; then, a similar idea is used to describe the disturbance as we will see later.

To the best of our knowledge, one of the first attempts to formally define disturbance in QM was realized by Martens and de Muynck in a seminal paper of 1992 [36], where they propose understanding the disturbance in quantum measurements in terms of non-ideality, namely by comparing the result of a measurement described by a POVM [31] and an ideal measurement. They proposed as a measure of disturbance (considered as a class of non-ideality) the Conditional Entropy (4)

(28) $$S\left(B|A\right)=-\sum_{a\in A,b\in B}p\left(a,b\right)\log\left(\frac{p(a,b)}{p(a)}\right).$$

This quantification of disturbance is viewed in terms of loss of information, an idea that was discussed in the previous section.

An interesting article by Appleby shows a general view of the concept of error (noise) and disturbance in its time [57]. The concepts of predictive and retrodictive probabilities are deeply studied. The work of Appleby is based on the articles by Arthurs and Kelly [58] and Arthurs and Goodman [56]. Here, the disturbance is measured by using disturbance operators for position and momentum:

(29) $$\begin{array}{ccc} \Delta_{x} & = & x_{f}-x_{i},\\ \Delta_{p} & = & p_{f}-p_{i}, \end{array}$$

for $x_{i},p_{i}$ the initial position and momentum, and $x_{f},p_{f}$ the final position and momentum operators. Here, the disturbance is measured as the root mean square of the disturbance operators

(30) $$D_{x,p}=\sqrt{\langle \psi ⊗\phi_{ap}\left|\Delta_{x,p}\right|\psi ⊗\phi_{ap}\rangle },$$

where |ψ〉 is the initial state of the system and $|\phi_{ap}\rangle$ the initial state of the measurement apparatus. The Heiseinberg picture is used in this article also. Additionally, it is important to say that the way in which the disturbance is measured is similar in nature to the one related to the original point of view of Heisenberg [10].

On the other hand, one of the best known articles about the concept of disturbance was published by Ozawa in 2003 [59]. In this work, the disturbance is treated as a mean value in the Heisenberg picture as follows:

(31) $$\eta \left(B,\psi ,A\right)={\left(\langle \psi |{\left(B^{out}-B^{in}\right)}^{2}|\psi \rangle \right)}^{1/2},$$

where *B* is an observable of the system, ψ the state of the system, A represents the measurement apparatus and $B^{out}$ and $B^{in}$ the *B* observable represented in two different times before and after the measurement of *A* with the apparatus A. It is worth mentioning that this proposal uses an ancilla in the model of measurement [60], a tool related to the generalized measurement formalism [31,61] but nowadays commonly used in the development of topics such as uncertainty relations or quantum information theory.

Otherwise, Branciard explores in a 2013 article [62] a way to study the uncertainty principle by using as a premise the original idea of Heisenberg. The work done in this paper consists in approximating a joint measurement of two observables of a system, even if the observables are incompatible. The author follows the work of Ozawa closely [59]; in this way, the disturbance is defined as the root mean squared deviation,

(32) $${\langle \psi ,\xi |B_{a}-B⊗I|\psi ,\xi \rangle }^{1/2},$$

in Equation (32), $B_{a}$ is an approximation of *B* which fullfils $[A_{a},B_{a}]=0$. The approximation of *B* is obtained from taking $B_{a}$ in an extended Hilbert space, where |ψ〉 is the state of the system and |ξ〉 is the state of a probe or ancillary system [60].

Following the framework established by Ozawa, Kaneda et al. [63] experimentally tested the NDT proposed by Ozawa [59] and Branciard [62] in generalized polarization measurements by utilizing weak measurements. For more details, see [63]. We emphasize that this work seems to be experimental evidence of Ozawa’s ideas; however, it must be recalled that this approach faces the constraint imposed by the Bell–Kochen–Specker Theorem [9,22,23].

On the other hand, an article by Lu et al. [64] studies the NDT relations and proves that it is possible for the total error in a measurement to be decomposed into two terms. These two terms are the operation bias and the fuzziness. See Reference [64] for a complete treatment. In this way, they obtain a stronger error trade-off than the proposed by Ozawa [59]. The disturbance is measured as the root mean square of a disturbance operator, taking the idea of [59]. In addition, the authors propose a circuit for testing their NDT, which adds to the work a complete description of the topic.

Moreover, an article by Mandayam and Srinivas from 2014 [65] is of considerable interest because they propose a different approach to the NDT. The main idea of this article is to measure the noise and disturbance by using probability distributions instead of observables [59,62]; consequently, they express the distinguishability of states by comparing the probability distributions before and after the measurement. Curiously, the two measures of disturbance that they proposed, which are based on the fidelity and the trace distance [25], are regarded as functions of the probability distributions. In this way, this article proposes the study of the NDT by means of a seminal idea, which will be exploited by other authors later [21,66,67].

Following the studies of Mandayam and Srinivas [66], they describe the NDT relations using the Tsallis Entropy as a measure of disturbance,

(33) $$T\left(p_{i}\right)=\frac{1}{1-\beta }\left(\sum_{i}p_{i}^{\beta }-1\right).$$

In Equation (33), $p_{i}$ is a probability distribution and β>0 a real constant different from 1. The authors demonstrate that the entropy of Tsallis is equivalent to the fidelity based measure of the disturbance for pure states [66]. In this case, the disturbance is characterized by comparing probability distributions as we see in [65].

Furthermore, Coles and Furrer proposed a disturbance measure in terms of the family of Relative α-Renyi entropies [67],

(34) $$D_{\alpha }\left(P|Q\right)=\frac{1}{\alpha -1}\log\left(\sum_{i}P_{i}^{\alpha }Q_{i}^{1-\alpha }\right),$$

where α∈[1/2,∞] and P,Q are two probability distributions. This work is a generalization of the NDT for a wider variety of probability metrics such as the relative entropy or the fidelity. This study implements the idea of distinguishability of states that uses the probability distribution before and after the measurement.

It is worth mentioning that a 2003 paper by Ozawa [59] has been a milestone in the development of uncertainty relations because the concepts of noise and disturbance proposed by him were not widely explored previously, although, as we mention above, Arthurs has used the noise operator before [56,58]. There are many works which take the idea of Ozawa’s article as a starting point for studying several ideas and scenarios.

Among the works which are influenced by this paper, we have an article by Dressel and Nori [13]. Here, we find a complete reformulation of Ozawa’s work [59], but taking into consideration the BKST. We must also mention that they study other articles besides [59], however their main description is based on this one. Additionally, the article is constructed with demonstrations for the incorrectness of distinct definitions of noise and disturbance due to the violation of the BKST. The main proposal of Dressel and Nori is to quantify the noise and disturbance by the implementation of single outcome measurements and the concepts of retrodictive and interdictive measurements, which are related to a priori and a posteriori probabilities. Here, the disturbance is defined as

(35) $$\eta_{B,k}={\left[\sum_{b,b^{\prime }}\left(B_{b}-B_{b^{\prime }}\right)p\left(b,b^{\prime }|k\right)\right]}^{1/2},$$

for an observable *B* and a single measurement of other observable *A*, whose outcome is *k*. The complete description of these quantities requires definitions and concepts that are properly explained in the original paper, however those are beyond the treatment of our work. We will only mention that $\left(B_{b}-B_{b^{\prime }}\right)$ is a function of the commutator between *B* and the measurement operators related to a priori and a posteri probabilities that are given in terms of the value $b^{\prime }$, which is the possible value after the outcome *k* of *A* and $p(b,b^{\prime }|k)$ is the conditional probability of the joint probability distribution given the outcome *k*. The main difference between (31) and (35) is that, while the former expresses the disturbance like a change between observables, the latter sets forth the disturbance in terms of state changes.

Ozawa himself improved his previous work in 2014 with the collaboration of Buscemi, Hall and Wilde [14]. The main idea of this article is to study the noise and disturbance by means of different types of loss of correlations in the framework of IT, i.e., in an information theoretic-approach, hereby, the lack of fulfillment of BKST is dismissed. This article provides a definition of disturbance related to the irreversibility of a measurement, given by the following expression

(36) $$D\left(M,Z\right)=\min_{\epsilon }H\left(Z|\hat{Z}\right),$$

where ϵ is an error correction operation, M an apparatus and *Z* an observable, $H(Z|\hat{Z})$ is the conditional entropy given by Equation (6) for the joint probability distribution, where $p\left(\hat{z},z\right)=p\left(z\right)p\left(\hat{z}|\psi^{z}\right)$, and p(z) and $p(\hat{z}|\psi^{z})$ are the a priori probability of observable *Z* and the conditional probability of observable *Z*, respectively, given an initial state $|\psi^{z}\rangle$, for estimating the value $\hat{z}$ adequately.

In this line of investigation, Sulyok et al. [68] describe a method for testing the improved proposal for the NDT of Ozawa [14]. The authors show the experimental validity of the relation for the qubit measurements. In this case, they utilized spin −1/2 neutrons to conduct the work. We want to emphasize the importance of this paper, considering the fact that it is crucial to have an experimental validation of the theory, which is necessary to complete an acceptable explanation of physics phenomena. Additionally, interesting experimental schemes were implemented by Iinuma et al. to test the error in the measurement outcomes by using photon pairs [69,70].

On the other hand, another interesting article is a recent paper by Benítez Rodríguez et al. [21], where a proposal is given in terms of an entropic uncertainty relation for disturbances of two observables of a system, the disturbance measure is related with the Jensen Shannon Entropy (9), and takes the following form

(37) $$D_{PQ}^{B}=\sqrt{\sum_{i=1}^{N}(p_{i}\ln\frac{2p_{i}}{p_{i}+q_{i}}+q_{i}\ln\frac{2q_{i}}{q_{i}+p_{i}}}),$$

where $p_{i}$ and $q_{i}$ are two probability distributions, associated with a priori and a posteriori probabilities of a given observable *B* due to a projective measurement of another observable *A*. As we can see, this definition is formulated by relating probability distributions, and using the distance between two probability distributions, an idea showed by Werner [71], but also parallel to the concept of distinguishability of states [72].

### 4.2. Precision–Disturbance Trade-off

To the best of our knowledge, Hofmann presented one of the earliest studies for the case of the original Heisenberg’s idea of precision–disturbance relation [73], i.e., that the resolution of measuring an observable *M* causes a disturbance on another observable *B*. To define disturbance, Hofmann firstly defines a measurement error as $\Delta B_{mf}^{2}=\delta B_{mf}^{2}+{\left(B_{f}-B_{mf}\right)}^{2}$, where $\delta B_{mf}^{2}$ is the random that limits the possibility to estimate *B*, and $B_{f}$ is the output value of *B* and $B_{mf}$ is the best possible estimate. Then, the averaged disturbance $\Delta B_{m}^{2}$ of *B* associated with the result *m* when measuring the generalized observable *M* is given by:

(38) $$\Delta B_{m}^{2}=\sum_{B_{f}}w_{m}\left(B_{f}\right)\Delta B_{mf}^{2},$$

where

(39) $$w_{m}\left(B_{f}\right)=\frac{\langle B_{f}|M_{m}M_{m}^{†}|B_{f}\rangle }{tr\{M_{m}M_{m}^{†}\}}.$$

Here, the disturbance $\Delta B_{m}^{2}$ can be associated with the loss of information as in previous sections. A key feature of the Hofmann’s analysis is the characterization of the uncertainties in terms of the physical properties of the system. That is, the measurement outcome *m* can be associated with the measurement of the observable *M* when the result $M_{m}$ was obtained, and the resolution $\delta M_{m}^{2}$ is attained [73]; this idea was extended to test entanglement [74]. In successive papers, Hofmann developed a significant work around this idea; for example, he found that the change of the density operator due to the measurement process can be expressed in terms of a dephasing factor $\eta =1-P_{M}$, where $P_{M}$ corresponds to the relative frequency of back-action disturbance in the sequential measurement of two non-commuting observables [75]; see also [76]. The relation between measurement outcomes and the physical properties of quantum systems is studied in Reference [77].

On the other hand, a recent proposal by Busch et al. defines ΔQ as the accuracy for position measurement and ΔP as the momentum disturbance incurred by measuring the position [78]. Conceptually, this approach shows some similarities with the approach of Srinivas for successive measurements [5,53,54,55]. Busch et al. also define the precision as the error that occurs when measuring *P*, i.e., the difference between the real value *P* and the value measured $P^{\prime }$. To define disturbance in this work, the authors take the root mean square deviation between an arbitrary distribution and a sharp value ξ. That is, $D={\langle {\left(p^{\prime }-\xi \right)}^{2}\rangle }^{1/2}$. Then, the disturbance as a measure of the error in a successive measurement of *Q* and *P*, is defined as $\Delta P=\lim_{\epsilon \to 0}\sup\left\{D|\rho ,\xi ;D\leq \epsilon \right\}$.

## 5. Conclusions

The concept of disturbance is commonly related to the uncertainty principle of quantum mechanics through the well-known statement: when one makes a measurement of the position, then the momentum is disturbed. However, the disturbance in quantum mechanics is more complex and versatile than what it is stated in many textbooks of quantum mechanics [11,21].

In-depth studies on the uncertainty principle have given birth to definitions of disturbance closely related to a wide variety of measurement frameworks. The measurement in quantum mechanics has a great significance, either theoretically and experimentally; therefore, it is expected to have such variety of definitions of disturbance as ways of implementing a quantum measurement in different scenarios. As we have previously showed in this paper, the definition of disturbance strongly relies on the context of the system that is analysed [20], saying it in a rough manner, it relies on the path that is chosen to measure it.

The disturbance is also studied alongside the information [7,34], thus setting the famous trade-off relations between the information gain and disturbance. Such has been the impact of the trade-off relations that the information gain–disturbance principle [7,12] is considered at the same level of importance as the uncertainty principle [7,12]. In addition, the trade-off relations between noise and disturbance are commonly studied as a different view of the uncertainty principle [14,21,59]. The previous branches of study provide new ways to conceive the disturbance in quantum systems and it gives theoretical and experimental originality to QM.

## Abbreviations

The following abbreviations are used in this manuscript:

QM	Quantum Mechanics
BKST	Bell–Kochen–Specker Theorem
IT	Information Theory
IDT	Information–Disturbance Tradeoff
MDPI	Multidisciplinary Digital Publishing Institute
DOAJ	Directory of Open Access Journals
TLA	Three Letter Acronym
LD	Linear Dichroism
